# National Insurance: A Warning from Germany

**Published:** 1912-11-23

**Authors:** 


### NATIONAL INSURANCE: A WARNING FROM GERMANY.

### The Lesson of Leipzig.

Dr. Gibbon has done excellent service in placing before the medical profession in this country a full *résumé* of the relations between the insurance societies and the doctors in Germany. It is an account which deserves careful study, for it clearly shows that nothing is to be gained by obscuring issues that must be faced resolutely or tampering for the time being with grave questions that must be peremptorily settled. The case, as Dr. Gibbon gives it in his recently published "Medical Benefit in Germany and Denmark," * stands as follows:

### Societies and Doctors at War.

When in 1904 the Leipzig insurance societies, with a membership of 140,000 insured persons, reviewed their position they found that they had contracts with 233 doctors whom they paid at the rate of 4s. 6d. per member per year, this sum assuring medical attendance to dependants of members. The doctors, on their part, claimed 12s. per member with dependants, free choice of doctor, new contracts, and as an indispensable preliminary to all agreements that these should not be terminable at the will of the society. To this demand the societies retorted by offering 5s. 6d. per member as a capitation fee. As the doctors were obdurate and well organised, negotiations were broken off and the societies took independent steps. They formed a staff of doctors, each of whom was guaranteed £300 a year with the privilege of private practice; they established three consultation clinics supervised by whole-time specialists paid at the rate of £400 per year, and they provided for the treatment of dependants by guaranteeing to pay the minimum State tariff for private treatment. The doctors, in retaliation, boycotted their colleagues in the service of the societies, and some of them refused to treat dependants of insured members on any terms. In the circumstances the societies found themselves severely handicapped, and for a time the dependants' benefit was dropped. The central authority finally intervened, deciding that a staff of 112 doctors was absolutely necessary to ensure efficient treatment, and steps were taken to make an agreement with the profession.

Under the German Insurance Act the central authority has full power to deal with such a contingency and may override, at its discretion, the decisions of the societies' executive. The new agreement stipulated for free choice of doctor, payment at the rate of 5s. per member without dependants and 3s. per dependant entitled to benefit, for special fees in certain cases, and for termination of the contracts with the societies' doctors at the earliest legally possible time. Provision was also to be made for controlling committees which could be appealed to in case of any professional difficulty. This agreement, distasteful enough to the societies, was ratified on appeal and remained in force until 1910. The societies, however, managed to reduce considerably the amount paid in capitation fees, so that the organised doctors found that they gained very little after all, since the salaries of the staff doctors had first to be paid out of the capitation fees, leaving a bare £10,000 per year for distribution among all the other medical men. The society doctors, too, formed special dispensary centres at which treatment was given for a small fee to dependants, so that in this way, too, the interests of the outside doctors were interfered with. The organised doctors fought hard and the societies found their consultative centres so costly that they were forced to close them. After some months of struggle friendly intervention succeeded in finding a *via media* and a new agreement was drawn up which is to run until 1916. By the terms of this compromise the doctors secured the principle of free choice of doctor, but yielded their comparatively high demand of pay, a much lower figure being settled. Dr. Gibbon gives full translations of the various agreements, which make instructive reading and which should be carefully perused by every medical man in this country before he decides his line of action in regard to the medical service under the National Insurance Act.

The Leipzig episode has shown that the interests of the societies and the doctors are much more closely allied than many members of the profession might be disposed to admit at first sight. It has also shown that it is quite possible to find a reasonable *modus vivendi* that shall be equitable to both sides without unduly straining the resources of the societies or overworking and underpaying the doctors. In discussing the matter it is worth while to point out that in the German Act there is no definite minimum laid down for medical benefit, nor is the amount distributable to the doctors arbitrarily fixed by the central authority. The societies have power to make their own terms with the profession, provided always that they stick to the general rules for the administration of medical benefit as drawn up by the central authority. Nor must it be forgotten that the profession in Germany has added help in the shape of numerous legal decisions bearing on the administration of such benefit that show clearly how willing the courts are to safeguard the just interests of the medical attendant. We have already quoted some of these decisions in previous articles on the German system that have appeared in these columns, but it is worth while to refer once more to the fact that such decisions exist.

### A Court of Professional Appeal.

Here it is still a debatable point what is sickness under the Act, and no doubt in practice considerable difficulty will be experienced in settling disputes on this score of essential interpretation. One thing the German societies have rigidly insisted upon, and that is that they must be the controlling authority in matters of administraton. To the professional mind there is usually a prejudice in accepting a society as master; the average doctor hardly likes

* Published by P. S. King and Son. Price 6s. net.

to be called a servant of the society that pays him. Yet if a doctor decides to join a panel he becomes an employee, and as such it is only just that he should fall into line with his fellow-employees, providing always that his expert work will be judged not by the layman, but by his professional peers. The controlling committees established under the Leipzig agreement provide a court of professional appeal which is of great utility, since they take into consideration all interests and are competent to deal with all disputes. To the profession in England the study of the German situation is both interesting and instructive, since it reveals that in co-operation and amicable compromise lies the best means of securing an adequate realisation of the promise of social betterment which the passing of the Insurance Act holds out. The strike there has failed, as it has failed in industrial and trade disputes, and whatever gain the profession or the societies have secured has been obtained by a judicious give-and-take policy that has been settled at round-table conferences. A similar settlement should not be impossible in this country, for however fully we all may agree that the Act needs amendment in some respects, only a fair trial can prove in how far the clauses relating to sickness benefit should be modified in the interests of the community at large.

